# Supplementary material for: Exploring gender differences in HIV-related stigma and social support in a low-resource setting: A qualitative study in the Dominican Republic
Source: PLoS One. 2023 Aug 24;18(8):e0290228. doi: 10.1371/journal.pone.0290228 (PMC10449144; doi:10.1371/journal.pone.0290228)
Supplement: Attachment — Submitted filename: Leonard Review of PONE-D-22-26942 .docx [file pone.0290228.s002.docx]

This is a very well-written qualitative study that documents an understudied topic -- the multiple levels of stigma experienced by HIV-infected women and men in the Dominican Republic and the gendered means by which individuals cope with stigma. The study has numerous strengths. It documents some unique differences between the stigma-related experiences of women and men. It uses the HIV Stigma Framework to both conceptualize the multiple levels of stigma and analyze the data. The authors wisely chose not to use the word stigma and discrimination in their interview guide although an explanation and/or citation as to why they chose to ask these questions indirectly was not provided. Another strength of the manuscript is the detailed description of the analysis strategy which is helpful for evaluating validity. The discussion section provides an excellent summary of the findings including suggestions for future research and specific intervention strategies. Table 2 is a helpful guide to the themes that emerged and outlined within the HIV Stigma Framework.

There are several weaknesses that dampen enthusiasm for the manuscript. The first weakness is that the introduction provides very little context regarding the HIV epidemic in the DR (e.g., epidemiology, ART availability, uptake, and viral suppression) as well as the context of the clinics -- that is, the manuscript indicates that they were purposively selected based on input from local partners but what was their input, how/why were these clinics selected? Providing more context would help the reader have a greater understanding of the quotes provided and their interpretation.

An additional weakness is the lack of description of the interviewers' and other analysts' positionality with respect to the study aims and the ways in which the researchers' experience shapes both the interviews and interpretation. A positionality statement may be particularly important for the research question and the context of this study and moreover, adds to the validity of the analyses. Further, a positionality statement may be particularly important for cross-cultural research teams.

Further, while the authors did an excellent job interpreting the data within the theoretical framework, the theoretical frame and resulting analysis essentially remains at the level of individual and thus marginally advances the literature on HIV-related stigma. The authors mention the intersectional nature of stigma but do not explicitly articulate how contextual social structures, institutional barriers, and historical events manifest themselves in the lived experiences described in the analysis for both women and men (and may be unique to the DR).

Finally, the authors do not report demographic information on sexual orientation or involvement in sex work which have been documented in the stigma literature in the DR.
