# Supplementary material for: Exploring gender differences in HIV-related stigma and social support in a low-resource setting: A qualitative study in the Dominican Republic
Source: PLoS One. 2023 Aug 24;18(8):e0290228. doi: 10.1371/journal.pone.0290228 (PMC10449144; doi:10.1371/journal.pone.0290228)
Supplement: Attachment — Submitted filename: PLOS ONE Review 01.16.23.docx [file pone.0290228.s003.docx]

PLOS ONE Review 01.16.23

OVERALL:

This is a robust paper that describes stigma among men and women living with HIV in the Dominican Republic. This manuscript is developed from secondary analysis of data from a parent study, where stigma was discussed indirectly. The authors conducted a content analysis based on pre-existing categories, guided by the HIV stigma framework. The authors describe similar and different ways that three types of stigma manifest in men vs. women. The manuscript adds to the HIV stigma literature by offering a gender-stratified lens, although it is unclear to this reviewer if this area of research is, in fact, previously unexplored.

This reviewer questions the overall soundness of the study findings based on questions of methodological rigor and analysis, highlighted by reporting most results in unsubstantiated quantitative terms. If the editors find this article suitable for their readership, I recommend a major revision with deep re-analysis to insure rigor, reporting and therefore updated discussion.

Background:

Authors provide helpful descriptions of various types of HIV related stigma and how stigma has been found to impact a range of socioecological levels of influence on peoples’ health and well-being. Identifying the HIV stigma Framework (lines 75-87) as a guiding conceptual framework is a strength of this paper.

The authors should revisit their presentation of the state of knowledge regarding the way HIV stigma has already been found to “be experienced differently by women compared to men”. (lines 88-97) This feels overstated, and can lead the reader to question why this analysis is being conducted in the first place, if the reasons for and prevalence of gender-based stigma are already known.

Last, the authors do make the case for comparing gendered experiences of HIV related stigma. However, they do not make the case (not even mentioned) for exploring experiences of social support and coping mechanisms. These factors need a rationale for why they are being studied. This is a theme throughout – it feels like social support and coping mechanisms were tacked on to the analytic plan without sufficient rationale or attention throughout.

Methods:

The authors claim to have analyzed the overall dataset ‘by gender’ but do not provide sufficient detail on their study design to support a gender-based analysis. First, more description is needed about recruitment (lines 157-8) of a ‘gender-balanced’ sample. How did you balance the sample? How many genders were included? How was gender identified to ensure the sample was ‘balanced’? did you exclude people of non-binary gender? In lines 172-3, gender options include ‘male, female’, which are labels for sex, not gender. This should be identified as a limitation, since it may have led to misclassification in the sample. Also, authors should indicate how they approached the gender analysis. Were the data analyzed separately by gender from the outset? Vs. Were the data analyzed as a full dataset and then segmented by gender? At what point did the analyses diverge?

I also have questions about the type of analysis that was conducted. From the authors’ descriptions, it appears that they in fact conducted ‘deductive’ content analysis, since they categorized their data by a pre-determined set of categories, according to categories within the HIV stigma framework ((ie experienced, anticipated, internalized) lines 189-200 ) and other a priori categories (ie social support).

In order to increase trustworthiness of the qualitative findings, I encourage the authors to follow COREQ guidelines to ensure they have included standard and sufficient info for the reader to determine trustworthiness in this manuscript. One example of a missing piece that should be included (when COREQ guidelines are followed) is description of any reflexive practices that occurred within the research team. This is just one example of what appears to be missing, but the article needs a thorough review to ensure other COREQ criteria are met minimally.

Results:

Where numbers are characterized (more, some, many, etc), please also include the numerator and denominator for each piece of information so that readers are clear of the proportions. This is crucial in qualitative work where it is not necessarily a given that data is self-initiated or prompted, or queried within the entire sample. It is noted that most of the results are reported in this enumerated fashion, which is not the intention of qualitative findings in general. Further, descriptions of one participant’s experiences are OK to describe, but it is preferable that the authors contextualize why they are exemplifying one participant rather than presenting the data as exemplary of a particular theme.

The analysis could be strengthened by deeper exploration of themes – there was lack of detailed thick description, which readers expect to see in qualitative reports. (p. 14&15, lines are not present) Instead the overwhelming reporting relies on quantitative comparisons of theme prevalence across men and women rather than identifying the qualitative characterization of men and women’s experiences. This makes the data read as potentially cherry-picked by the authors, and calls into question if there is additional, different, or more mature analysis to be done. I recommend conferring with a qualitative methodologist to examine these issues.

Table 1 –

Age is reported as a mean, but no standard deviation is reported as stated. Please make consistent. The ‘occupation’ category is confusing. These are not mutually exclusive categories as described For example, one can be self-employed, but only employed part-time. Can the authors clarify this? Additionally, please define Food Insecurity status.

Discussion:

Due to methodological shortcomings and questions over the robustness and trustworthiness of the analysis, I am refraining from commenting on the Discussion section.
