# Supplementary material for: Exploring gender differences in HIV-related stigma and social support in a low-resource setting: A qualitative study in the Dominican Republic
Source: PLoS One. 2023 Aug 24;18(8):e0290228. doi: 10.1371/journal.pone.0290228 (PMC10449144; doi:10.1371/journal.pone.0290228)
Supplement: Attachment — Submitted filename: Gender and Stigma_ Response to Reviewers_final 05-23-23.docx [file pone.0290228.s004.docx]

**Response to Reviewers**

**PONE-D-22-26942**

**Exploring gender differences in HIV-related stigma and social support in a low-resource setting: A qualitative study in the Dominican Republic**

Thank you for the opportunity to revise and resubmit our manuscript (PONE-D-22-26942). We appreciate the suggested revisions and the reviewers’ attention to detail. We have addressed all reviewer comments (see below) and believe this manuscript is stronger because of the reviewers’ thoughtful comments and suggestions.

**Reviewer 1**

1. **This is a very well-written qualitative study that documents an understudied topic -- the multiple levels of stigma experienced by HIV-infected women and men in the Dominican Republic and the gendered means by which individuals cope with stigma. The study has numerous strengths. It documents some unique differences between the stigma-related experiences of women and men. It uses the HIV Stigma Framework to both conceptualize the multiple levels of stigma and analyze the data.**

**Response**: We thank the reviewer for their comments and careful review of the paper.

2. **The authors wisely chose not to use the word stigma and discrimination in their interview guide although an explanation and/or citation as to why they chose to ask these questions indirectly was not provided.**

**Response**: We thank the reviewer for this comment. We added text in the manuscript (see page 9) to explain how and why we asked about these questions more indirectly. Briefly, the data for this analysis were drawn from a formative research study conducted primarily to inform the development of a food security intervention for people with HIV and adherence difficulties. We did have a section on “Experiences with HIV,” where we asked about their experiences with diagnosis, physical and mental health changes, social relationship changes, social support, feelings of guilt or shame, and whether they have been victims of verbal or physical violence. Our intent was for these narratives about stigma and social support to emerge more naturally from questions about their experiences with HIV.

3. **Another strength of the manuscript is the detailed description of the analysis strategy which is helpful for evaluating validity. The discussion section provides an excellent summary of the findings including suggestions for future research and specific intervention strategies. Table 2 is a helpful guide to the themes that emerged and outlined within the HIV Stigma Framework.**

**Response**: We thank the reviewer for these comments.

4. **There are several weaknesses that dampen enthusiasm for the manuscript. The first weakness is that the introduction provides very little context regarding the HIV epidemic in the DR (e.g., epidemiology, ART availability, uptake, and viral suppression) as well as the context of the clinics -- that is, the manuscript indicates that they were purposively selected based on input from local partners but what was their input, how/why were these clinics selected? Providing more context would help the reader have a greater understanding of the quotes provided and their interpretation.**

**Response**: We addressed these comments by doing the following: 1) In the background (on pages 4-5), we added new information about the adult HIV prevalence rate, ART availability, ART uptake, and viral suppression in the Dominican Republic; and 2) on page 8, we provide more explanation around the selection of these clinics for the overall study, namely, the clinics were located in provinces with the country’s highest HIV prevalence, in comparable regions, and sufficiently distant from one another to avoid cross-contamination.

5. **An additional weakness is the lack of description of the interviewers' and other analysts' positionality with respect to the study aims and the ways in which the researchers' experience shapes both the interviews and interpretation. A positionality statement may be particularly important for the research question and the context of this study and moreover, adds to the validity of the analyses. Further, a positionality statement may be particularly important for cross-cultural research teams.**

**Response**: We added a paragraph with information about author positionality on page 7.

6. **Further, while the authors did an excellent job interpreting the data within the theoretical framework, the theoretical frame and resulting analysis essentially remains at the level of individual and thus marginally advances the literature on HIV-related stigma. The authors mention the intersectional nature of stigma but do not explicitly articulate how contextual social structures, institutional barriers, and historical events manifest themselves in the lived experiences described in the analysis for both women and men (and may be unique to the DR).**

**Response**: To address this comment, more information about the context of living with HIV in the Dominican Republic has been added to both the Introduction and Discussion, most notably on pages 4-6 of the introduction, where the role of poverty, limited availability of health care resources, and low social support are mentioned. In the Results, respondents’ descriptions reflect broader social attitudes and norms while revealing the layers of marginalization experienced by people with HIV. These responses reflect not only individual experiences, but also the social, political, and economic context in which they occur (Bowleg, 2008). In the Discussion, we elaborate on the drivers of gender violence in the Dominican Republic, which include poverty, discrimination, intersectional stigma, and machismo (pages 28, 29, and 32).

7. **Finally, the authors do not report demographic information on sexual orientation or involvement in sex work which have been documented in the stigma literature in the** **DR.**

**Response**: We did not collect this information and have added this to the limitations (see pages 31-32).

**Reviewer 2**

1. **This is a robust paper that describes stigma among men and women living with HIV in the Dominican Republic. This manuscript is developed from secondary analysis of data from a parent study, where stigma was discussed indirectly. The authors conducted a content analysis based on pre-existing categories, guided by the HIV stigma framework. The authors describe similar and different ways that three types of stigma manifest in men vs. women. The manuscript adds to the HIV stigma literature by offering a gender-stratified lens, although it is unclear to this reviewer if this area of research is, in fact, previously unexplored.**

**Response**: We thank the reviewer for these comments about our manuscript adding to the HIV stigma literature. To address the comment about whether this area is previously unexplored, we did several things to provide more information in the Introduction about this study’s contributions to the literature: 1) we clarify that there is some evidence in the literature about gender differences in stigma experiences (see next point and page 4 of the manuscript); 2) we refer to literature specifically from the DR on stigmatization of homosexuality and bisexuality (see page 5) and stigma affecting engagement in HIV care of various populations (see pages 5-6); 3) we clarify that, to our knowledge, prior studies have not explored differences in HIV-related stigma and social support between men and women with HIV in the DR (see page 6).

2. **This reviewer questions the overall soundness of the study findings based on questions of methodological rigor and analysis, highlighted by reporting most results in unsubstantiated quantitative terms. If the editors find this article suitable for their readership, I recommend a major revision with deep re-analysis to insure rigor, reporting and therefore updated discussion.**

**Response**: We have revised the manuscript substantially to provide additional information on the methodological rigor and analytical approach (see additional text on pages 9-11, Data Collection and Data Analysis). Further, we have expanded the Results section to include more qualitative data (participant narratives) to demonstrate themes identified (see pages 13-27). Quantitative data are presented only for participant characteristics (as descriptive data from our sample). More details about the specific changes are provided under responses to #6, 7, 8, and 9 below.

3. **Authors provide helpful descriptions of various types of HIV related stigma and how stigma has been found to impact a range of socioecological levels of influence on peoples’ health and well-being. Identifying the HIV stigma Framework (lines 75-87) as a guiding conceptual framework is a strength of this paper.**

**Response**: We thank the reviewer for these comments.

4. **Background: The authors should revisit their presentation of the state of knowledge regarding the way HIV stigma has already been found to “be experienced differently by women compared to men” (lines 88-97). This feels overstated, and can lead the reader to question why this analysis is being conducted in the first place, if the reasons for and prevalence of gender-based stigma are already known.**

**Response**: The reviewer is correct that we overstated this. We revised this statement to read, “There is some evidence that being diagnosed with HIV and resultant HIV-related stigma may be experienced differently by women compared to men” (page 4).

5. **Background: Last, the authors do make the case for comparing gendered experiences of HIV related stigma. However, they do not make the case (not even mentioned) for exploring experiences of social support and coping mechanisms. These factors need a rationale for why they are being studied. This is a theme throughout – it feels like social support and coping mechanisms were tacked on to the analytic plan without sufficient rationale or attention throughout.**

**Response**: We thank the reviewer for this comment. The questions on stigma and social support were both included under the interview guide heading on “Experiences with HIV,” thus we did plan to analyze social support all along. However, we do see that in the previous version, we failed to set this up appropriately in the Introduction. To better justify this focus, we added a paragraph on pages 5-6 that summarizes the literature on social support and coping among PLHIV in the Dominican Republic.

6. **Methods: The authors claim to have analyzed the overall dataset ‘by gender’ but do not provide sufficient detail on their study design to support a gender-based analysis. First, more description is needed about recruitment (lines 157-8) of a ‘gender-balanced’ sample. How did you balance the sample? How many genders were included? How was gender identified to ensure the sample was ‘balanced’? did you exclude people of non-binary gender? In lines 172-3, gender options include ‘male, female’, which are labels for sex, not gender. This should be identified as a limitation, since it may have led to misclassification in the sample. Also, authors should indicate how they approached the gender analysis. Were the data analyzed separately by gender from the outset? Vs. Were the data analyzed as a full dataset and then segmented by gender? At what point did the analyses diverge?**

**Response**: We corrected and added some information to address these comments. First, we added information (see page 8) about how we tried to recruit an equal number of men and women in the parent study. The gender options used in our sociodemographic questionnaire of participants were in fact “hombre” (man), “mujer” (woman), and “otro” (other; if selected, they were asked to specify their gender). All our participants selected either “hombre” or “mujer;” no one selected “other.”

In the earlier submission of our paper, we incorrectly referred to gender in the text on participant characteristics as “male” and “female” and have corrected that in this revised version to “man” and “woman” (see bottom of p. 9). We also added a statement in our limitations that these findings may not apply to other gender identities (see pp. 31-32).

In terms of how we approached the analysis, we provide more details in the revised version. Specifically, on page 10, we note that in coding of the data for the overall study, we did not consider gender. Also on pages 10-11, we note that once all data were coded and we undertook the qualitative analysis regarding gender differences in stigma and social support, we segmented the coding reports by gender and created matrices organized by participant and code, with one matrix for men and the other for women, allowing for constant comparison of patterns – including salience, range, convergence and divergence – by gender across participants and within codes.

7. **Methods: I also have questions about the type of analysis that was conducted. From the authors’ descriptions, it appears that they in fact conducted ‘deductive’ content analysis, since they categorized their data by a pre-determined set of categories, according to categories within the HIV stigma framework ((i.e., experienced, anticipated, internalized) lines 189-200) and other a priori categories (i.e., social support).**

**Response**: On pages 9-10, we have provided information to clarify our analytic approach. It was a combined process where we used predetermined categories from the interview guide (deductive) and allowed for emergent codes (more inductive).

8. **Methods: In order to increase trustworthiness of the qualitative findings, I encourage the authors to follow COREQ guidelines to ensure they have included standard and sufficient info for the reader to determine trustworthiness in this manuscript. One example of a missing piece that should be included (when COREQ guidelines are followed) is description of any reflexive practices that occurred within the research team. This is just one example of what appears to be missing, but the article needs a thorough review to ensure other COREQ criteria are met minimally.**

**Response**: We reviewed the COREQ guidelines and added information that was missing. We can confirm that our data analysis utilized a structured protocol with multiple qualitative researchers and reflexive practices. As described in our paragraph on Author positionality (page 7) our team represented diverse, intersectional identities and relevant professional and lived experiences. We also added text to the Study overview (pages 7-8) to emphasize the community-partnered nature of our study, which involved community partners in all phases of the research, including development of the research questions and interview guides, review of preliminary findings, and dissemination. We are attaching the completed COREQ guidelines form.

9. **Results: Where numbers are characterized (more, some, many, etc.), please also include the numerator and denominator for each piece of information so that readers are clear of the proportions. This is crucial in qualitative work where it is not necessarily a given that data is self-initiated or prompted, or queried within the entire sample. It is noted that most of the results are reported in this enumerated fashion, which is not the intention of qualitative findings in general. Further, descriptions of one participant’s experiences are OK to describe, but it is preferable that the authors contextualize why they are exemplifying one participant rather than presenting the data as exemplary of a particular theme.**

**Response**: We did several things to address this concern. First, we clarify in the paper that participants’ narratives are presented as exemplary of a particular theme (see page 11). Our intent was to provide a qualitative sense of the salience and range of experiences on any given topic and give a general sense of where men and women participants’ experiences converged (i.e., were felt similarly across several participants) vs. diverged (i.e., were less common). Merely counting and providing a numerator and denominator for the qualitative results would not be appropriate, as it would give a false sense of precision. We did retain the numerator and denominators for the description of participant characteristics and clarified that these were collected through a structured questionnaire (see pages 11-12). Second, we revised the text in various places throughout results (pages 13-27) to clarify that when we say, “one participant,” we are providing an illustrative quote to add context to the themes identified across various participants’ narratives. Finally, we provided additional explanation for some of the themes regarding the differences that emerged by gender.

10. **Results**: **The analysis could be strengthened by deeper exploration of themes – there was lack of detailed thick description, which readers expect to see in qualitative reports. (p. 14&15, lines are not present) Instead the overwhelming reporting relies on quantitative comparisons of theme prevalence across men and women rather than identifying the qualitative characterization of men and women’s experiences. This makes the data read as potentially cherry-picked by the authors, and calls into question if there is additional, different, or more mature analysis to be done. I recommend conferring with a qualitative methodologist to examine these issues.**

**Response**: We did several things to address this concern: 1) we provide more details on our data analysis approach to the methods section (pages 10-11, see answer to #4 above); 2) we added 18 additional quotations from our coded data to provide a deeper exploration and illustration of the themes (12 additional quotations on stigma themes and 6 additional quotations on social support); 3) we added descriptors to all the quotations (ID, age, and gender) to further illustrate the breadth of responses; 4) we moved Table 2, which summarizes the themes about the different kinds of stigma and compares examples and consequences between women and men, to later in the manuscript, after the presentation of results and participants’ narratives.

11. **Table 1 – Age is reported as a mean, but no standard deviation is reported as stated. Please make consistent. The ‘occupation’ category is confusing. These are not mutually exclusive categories as described. For example, one can be self-employed, but only employed part-time. Can the authors clarify this? Additionally, please define Food Insecurity status.**

**Response**: Standard deviation has been added to table 1. We asked participants to state their current primary occupation – we clarify this in Table 1 and in the text on pages 9 and 12. Finally, we also added an * to Food Insecurity Status in Table 1 to indicate that this was measured using the Latin American and Caribbean Food Security Scale (ELCSA) and provide a citation.

12. **Due to methodological shortcomings and questions over the robustness and trustworthiness of the analysis, I am refraining from commenting on the Discussion section.**

**Response**: As described above, we made extensive revisions to demonstrate the robustness and trustworthiness of our analyses, which support the points made in the Discussion.
