# Supplementary material for: Exploring gender differences in HIV-related stigma and social support in a low-resource setting: A qualitative study in the Dominican Republic
Source: PLoS One. 2023 Aug 24;18(8):e0290228. doi: 10.1371/journal.pone.0290228 (PMC10449144; doi:10.1371/journal.pone.0290228)
Supplement: Attachment — Submitted filename: Gender and Stigma_ Response to Reviewers R2.docx [file pone.0290228.s005.docx]

**Response to Reviewers**

**PONE-D-22-26942R1**

**Exploring gender differences in HIV-related stigma and social support in a low-resource setting: A qualitative study in the Dominican Republic**

Thank you for the opportunity to revise and resubmit our manuscript (PONE-D-22-26942R1). We appreciate the suggested revisions and the reviewers’ attention to detail. We have addressed all reviewer comments (see below) and believe this manuscript is stronger because of the reviewers’ thoughtful comments and suggestions.

Below we outline how we addressed reviewer’s concerns, using **bolded Calibri font** for the reviewer comments, regular (not bolded) Calibri font for our explanation, and Times New Roman font for text that was cut and pasted from the manuscript (the latter to show exactly how we addressed the comment in the manuscript).

**Reviewer 2**

1. **This article has been much improved since the revision and most reviewer comments have been addressed, although there is one outstanding concern that is a critical one:

   My previous review highlighted concerns about the trustworthiness of the qualitative analysis, because of key issues with the methods. The authors attempted to address my concerns by adding more quotes and providing more explanation for their analytic approach. While their changes did improve the robustness of their analysis, it did not address my biggest concern about the trustworthiness due to methodological shortcomings. If anything, their response conjured more concern about methodological appropriateness of the analytic method they used and, subsequently, how they report their findings.**

**Response**: We appreciate knowing that the changes we previously made to the manuscript have improved its quality, and we value your attention to detail. We added quotes to address the earlier comments about the need for a deeper exploration of themes. We agree that these additions improved the robustness of our analysis, which is often noted (e.g., COREQ guidelines) as a means for increasing trustworthiness of reported qualitative data. Considering the details provided in these suggestions, we have added information and detail to the manuscript to address additional concerns. Specifically, we:

A. Clarified that our approach was qualitative content analysis, which included both deductive and inductive approaches to coding (see answers to #2, 3, and 4 below).
B. We elaborated on the steps we took to increase the trustworthiness of the data collection and analysis processes (see pages 10, 11, and 12 and excerpts below). These included having multiple trained coders, utilizing field notes, the use of a collaboratively developed interview guide and code books, the use of memos during the coding process, and the use of representative quotes. See, for example:

(p. 10): Using the Consolidated Criteria for Reporting Qualitative Research (COREQ) guidelines, steps were taken to ensure the trustworthiness of the process of data collection and resulting data, including the collaborative development of instruments, providing training to interviewers, the use of field notes and an interview guide during interviews, and audio-recording (40).

(p. 11): Trustworthiness in this initial phase of data analysis was facilitated through the using memos during the coding process, developing an iterative codebook, engaging multiple independent coders, and resolving discrepancies via consensus (40, 46).

(p. 12): Trustworthiness in this phase was facilitated by using analytical memos, extensive discussion and constant feedback as a team (including with team members with lived experience), developing matrices with the themes identified in the code reports to further organize the data, and using representative quotations to illustrate themes and trends in the data (40, 46).

2. **In the previous and current manuscript versions, the authors state they conducted content analysis. First, it is not clear to me the rationale for using content analysis, since their research question does not fit squarely in the more typical types of research questions where content analysis is used (i.e., how topics are communicated/discussed, presence or absence of a theme, etc.). I could imagine a choice to use content analysis being made so the researchers could determine, for example, differences in theme presence or salience between the 2 gender groups. However, the authors specifically chose not to include any quantified analysis (no numerators/denominators) as I had offered as a possibility in my last review, and they do not explain how the meaning units/codes were determined and related to one another. It is not essential to present quantitative data here, but it is critical in content analysis that the data segments that were coded into meaningful units are in the very least well defined and described. The analytic process required for this is not presented, and therefore I assume was not conducted.**

**Response**: We clarify that we conducted qualitative content analysis and provide a rationale on p. 8:

The present study uses qualitative content analysis (34) to explore how PLHIV in the Dominican Republic experienced, anticipated, and internalized stigma and social support and how these experiences may have differed by gender. Qualitative content analysis was selected to address this study question due to the flexibility of utilizing multiple approaches (e.g., inductive and deductive) in order to derive meaning and to understand the phenomena in question (35). Further, content analysis is a method that is well studied and provides an effective means of organizing and deriving meaning and conclusions from textual data (35, 36).

In addition, we made extensive edits to our description of our methods under Data Analysis (pp. 10-12) to clarify our analytic process. Briefly, we developed a codebook using deductive codes derived from the interview guide while allowing for emergent codes (inductive). The codes used for the present paper are listed on p. 11. We also clarified that code reports (with all the participant narratives tagged for that code) were then analyzed and used to systematically document themes across participants and codes and then compared by gender (using matrices). See pages 10-12 for a full description of this process.

3. **Second, they omit important descriptions of the content analysis approach they did take, if in fact content analysis was used. They do not describe if it was a latent vs. semantic content analysis; conceptual vs. relational, etc...**

**Response**: We added detail about this on p. 12:

The qualitative data, at all points of this analysis, were examined for semantic (also known as manifest) meaning, without regard for substratum and latent meaning that can be interpreted in participant physical gestures, laughter, sighs, and silence, as these can oftentimes result in an over-interpretation of data (35, 46). The focus instead was on participants’ narratives tagged as relating to one of the codes relevant to the research questions (47).

4. **Third, the references for their methodological approach mainly refer to grounded theory rather than content analysis. So it leaves me further questioning the overarching methodology and subsequent findings, if in fact they conducted modified grounded theory, thematic analysis or some other approach. While possible that the term ‘content analysis’ is merely being misused or the methods are just not adequately described, either way this is a critical flaw in this manuscript and indicates the need for an additional or different analysis to be conducted.**

**Response**: We apologize for the confusion. Our earlier version did cite references for qualitative content analysis when we talked about theme generation while reviewing code reports, but we neglected to include them earlier in the Data Analysis section. We now cite the references for qualitative content analysis at the beginning of the Methods section (p. 8, as noted above under Response to #2), as well as when we talk about our process for coding:

(p. 10) Coding procedures for the full dataset (i.e., across all topics, not just stigma experiences, coping, and social support) consisted of qualitative content analysis methods (34) using deductive approaches while also allowing for emergent codes (41, 42) or inductive approaches.

34. Hsieh HF, Shannon SE. Three approaches to qualitative content analysis. Qual Health Res. 2005;15(9):1277-88.

(p. 10) This approach involved creating initial codes based on the interview guide, coding the data, then grouping similar codes to create categories (44, 45). In re-reading transcripts, coders wrote memos for text that did not adhere to the established coding structure, which were used to generate emergent codes. Qualitative content analysis has been identified as “unique” in being able to combine deductive and inductive approaches (35).

44. Hickey G, Kipping C. A multi-stage approach to the coding of data from open-ended questions. Nurse Res. 1996;4(1):81-91.

45. Krippendorff K. Content analysis: an introduction to its methodology. Beverly Hills, CA: Sage; 1989.

35. Cho JY, Lee E-H. Reducing confusion about grounded theory and qualitative content analysis: Similarities and differences. Qualitative Report. 2014;19(32).

5. **To start, here is a good article that helps describe the difference bw grounded theory/content analysis. I think articles like these (and/or qual methods texts) are helpful in understanding nuances in qual methods approaches:
   Cho JY, Lee EH. Reducing confusion about grounded theory and qualitative content analysis: Similarities and differences. Qualitative report. 2014 Aug 11;19(32).**

**Response**: We appreciate the reviewer recommending this article by Cho & Lee (2014), which we used to clarify our approach and cite several times in the revised manuscript. We have added details to the methods section to describe more fully and justify the use of qualitative content analysis (e.g., see p. 8 and answer to #2 above).
